# American Medical Association

**Published:** 1853-05

**Authors:** 


### THE WESTERN JOURNAL.

THIRD SERIES.—VOL. XI.—NO. V.

LOUISVILLE, MAY, 1853.

### AMERICAN MEDICAL ASSOCIATION.

The sixth annual meeting of the *American Medical Association* took place in the Bleecker Street Presbyterian Church, New York, on Tuesday the 3rd instant. The attendance was unusually large. On taking the chair, the President, Dr. Welford, congratulated the members of the Association on the happy return of the anniversary, the thronged attendance of delegates from all parts of the Union, and the flattering prospects and advancement of the profession. Dr. F. Campbell Stewart, as Chairman of the Committee of Arrangements and Reception, then came forward and addressed the Association in the following terms :

*Mr. President and Delegates*—At the commencement of its annual meeting, permit the physicians of New York to congratulate the American Medical Association upon the recurrence of its anniversary. Seven years have elapsed since the preliminary meeting of the convention which recommended the organization of this national congress assembled here; and as New Yorkers, we indulge in a feeling of proud satisfaction at the triumphant success which has attended so important a movement, originating in our State. The labors of the learned body over which you, sir, now preside, have been most arduous; but the result has not disappointed the expectations of the friends of reform and of progress in our profession. Mighty objects are aimed at; important achievements have been already accomplished and a guarantee is afforded of the eventual fulfilment of the desires of those whose aspirations for the advancement and perfection of medicine led them to propose the foundation of this association, which, representing the whole fraternity throughout our wide spread country, assembles annually for scientific discussion, and to consult upon matters pertaining to the general welfare of the entire faculty. The five published volumes of your "Transactions" afford abundant and conclusive evidence of the zeal by which they are actuated, and the ability which characterizes the scientific labors of your members. The meeting of this year has been generally anticipated with peculiar interest: an unusually large attendance has been expected; numerous papers will be presented; valuable reports are to be rendered, and much of your time will be required for the consideration of subjects of grave importance—hence I shall not now detain you longer than to assure you that your advent has been anxiously looked for by us, and your colleagues here have been desirous to manifest their appreciation of the cause in which you are engaged, and their estimation of the favor conferred by the Association in selecting this metropolis as the place for holding the present session. With the view of making suitable preparations for your reception and accommodation, they have delegated members from each of their societies and institutions, to co-operate with your Committee of Arrangements. It is, then, in the name of the united profession of New York, thus represented, that we tender you a sincere, a hearty, and a cordial welcome to our city.

The following table exhibits the representation of the States at this period of the proceedings:

| *States and Districts.* | *No. of Delegates.* | *States and Districts.* | *No. of Delegates* |
|---|---|---|---|
| New York | 132 | Ohio | 7 |
| Massachusetts | 44 | Indiana | 8 |
| Pennsylvania | 40 | Missouri | 6 |
| Maryland | 17 | North Carolina | 5 |
| New Jersey | 15 | South Carolina | 10 |
| New Hampshire | 11 | Georgia | 3 |
| Virginia | 14 | Alabama | 2 |
| Rhode Island | 12 | Iowa | 1 |
| Connecticut | 29 | Delaware | 2 |
| Kentucky | 5 | Maine | 4 |
| Michigan | 6 | Vermont | 6 |
| Illinois | 6 | District of Columbia | 5 |
| Tennessee | 3 | | |
| Total number of Delegates | | | 390 |

The annual address of the President was well received. It was elaborate, and in the main the views were such as we can heartily endorse; in some of his positions we cannot concur. He took a review of the history of the *status* of the medical profession in America from the early days of the republic—of the evils which rose from the competition of the infant colleges and chartered bodies, and the indiscriminate and ill judged issuing of charters by many of the State Legislatures. He congratulated the association upon the improvement which was now visible in these respects. He pressed upon the delegates the necessity which existed, professionally and philosophically, for exertion to secure an enactment rendering a uniform registry of marriages, births, and deaths, incumbent all over the land. The Legislature of Virginia had already set the example. The adulteration of drugs, both imported and of home manufacture, was well worthy their attention, and suitable State laws of prevention would be better than the federal act now in force.

Dr. Baillie, who was appointed Inspector at New York, made a startling statement relative to the fraud perpetrated in this way. Out of a million and a half of dollars worth of drugs entered at the Custom House, one half was useless or worthless. Rhubarb, jalap, iodine, and yellow bark were adulterated frightfully, and would have reached the stomachs and persons of the people but for the inspector. The number of talented papers and essays which had emanated from this body had tended to elevate the character of the profession in America, and afforded a complete answer to the assertions of some persons that the action of the body had the effect of propagating homœpathy and other delusions. The meeting here to-day was proof of the onward course of the profession, and practitioners from the crowded city and silent forest, from the frozen North and sunny South, from the mountain and the prairie, were before him, animated by a common anxiety for its honor and independence. The genius, the talent, and the energy of the profession should be concentrated by the organization of local and State societies. He would next allude to the present faulty mode in which licenses to practice medicine were granted, and the impunity with which men may assume the name of doctor, who are not endowed with talent for that high calling. A proper Board of Medical Examiners, having nothing to do with schools, should be instituted in each State. with power to demand that certificates of attendence upon a proper curiculum of study be produced by all candidates. This would improve the system of teaching in the schools as pupils would frequent schools affording the fullest conrse of instruction. In accordance with a resolution passed last year, he had deputed Dr. J. F. Lambe, of Pennsylvania, to report to the body upon medical matters when traveling in Europe. Resignations of members of committees upon epidemics had taken place in North Carolina and Virginia He had the melancholy duty to announce to the delegates that since they last met, death had been amongst them, and taken away Doctors Drake and Horner, two of the most brilliant stars of the association. As he was now retiring from the Presidency, he would, as if with dying words, conjure the delegates to unite with ardor for the honor and dignity of the noble profession which they graced. (Applause.)

The nominating Committee recommended the following officers for the ensuing year, and they were unanimously elected:

PRESIDENT.

Doctor Jonathan Knight....................Connecticut

VICE PRESIDENTS.

Doctor Usher Parsons.........................Rhode Island.

Doctor Lewis Condit........................New Jersey.

Doctor Henry R. Frost....................South Carolina.

Doctor H. L. Howard.......................Ohio

SECRETARIES.

Doctor E. L. Beadle........................New York.

Doctor Edwin L. Lamoine..................Missouri.

TREASURER.

Doctor D. Francis Condie..................Pennsylvania.

Upon motion of Dr. Hopkins, (Maryland,) it was resolved that no speaker shall engage the attention of the meeting for a longer period than ten minutes at one time upon one subject, and that he shall not speak a second time upon the same subject without the permission of the meeting previously obtained.

Doctor Campbell Stewart moved that the Association do meet at 9 o'clock to-morrow morning and sit until 12; that a recess of an hour be then taken, and, after reassembling, that the members sit from 1 to 4 o,clock in the afternoon.

The second day's session commenced at 9 o'clock A. M. The Association was called to order by the president, Dr. Jonathan Knight, of New-Haven. The minutes of last meeting were read by Dr. Beadle, Secretary, which were approved. A reconsideration was moved by Dr. Cox of the report of the committee of Arrangements, in order to allow Drs. Bache and Pinckney, of the U. S. Navy, to take their seats as delegates by appointment of the chief of their bureau, when the following modification of the former resolution was put and carried:

*Resolved,* That the representatives of the Army and Navy shall be admitted as delegates to this Association when appointed by the chiefs of their respective bureaus.

The delegates were then admitted.

New delegates were announced, and admitted to the floor.

A number of gentlemen of the profession were invited to be present.

The President announced that it was in order to hear the reports of the several Standing Committees. The Secretary then proceeded to call them in the order in which they were registered.

Prof. Meigs, of the Jefferson School, Chairman of Committee on the Diseases of the Cervix Uteri, desired that his Report might not be read, as there were many persons present who did not belong to the profession, and he thought the subject of his Report was of a nature exclusively professional. Report referred to the Committee on Publication.

Dr. Condie, of Philadelphia, reported on Tubercular Diseases, that he was sorry to be unable to present his Report at this meeting, owing to the multiplicity of duties which the Society had imposed on him at its last meeting. He had paid a great deal of attention to this class of diseases, and such was the extent of the subject for investigation, by microscopic and other modes, that it would occupy him until next year to report fully. On motion, Committee continued to next annual meeting.

Reports from Committees on various other subjects were called for but there was no response.

Dr. Emerson, of Philadelphia, presented his report on "The Agency of Refrigeration produced by upward radiation of heat as an exciting cause of Disease;" Dr. Haase, and Dr. Ruschenberger, of Philadelphia, colleagues. The annexed is a brief abstract: The effect of it on the system by sudden reductions of temperature from exposure to various atmospheric influences, among which is prominent; terrestial or upward radiation of heat, the nature and laws of which require to be more generally known, to be properly guarded against. The inhabitants of cities suffer less than those of the country. From radiation persons exposed to the chilling influences of upward radiation during the night, in malarious districts, will, in all probability, contract the prevailing endemic or epidemic; or in the absence of a predisposition to disease of any particular form, will have diarrhea, rheumatism, or some other disease. While soldiers in malarious districts, who have had but the slightest shelter, have been known to escape disease altogether, while those not so protected have suffered severely. The whole tendency of the Report is to show how an acquaintance with a single law of nature, may, by following out its relations, be made to shed a new light upon obscure subjects, and to lend to the correction of long-existing errors. The sanatory lesson designed to be inculcated is the great importance of gaurding against the refrigerating effects of *nocturnal radiation*, especially in sickly places and during epidemic periods.

Dr. Campbell, of Augusta, then reported on Typhoid Fever; the causes of this fever he traced to the lesions of the ganglionic system.

Dr. Atlee, of Lancaster, Penn., reported on the epidemics of New-Jersey, Delaware and Pennsylvania. He deprecated the difficulty of obtaining adequate cooperation in an investigation of this character; he was sorry that so much apathy was exhibited in a matter of such vital importance.

Dr. Sutton, of Kentucky, presented an abstract report on the epidemics of that State and Tennessee. Cholera, bilious fever, dysentery, typhus and typhoid fever, cholera infantum, and other diseases appearing in the different districts of the two States periodically, were treated under ten different heads.

Dr. Pitcher, of Detroit, Chairman of the Committee on Medical Education in the United States compared with Medical Education in other countries, reported.

They deprecated the prevalence of quackery in the United States, although somewhat diminished since the formation of this Association, and strongly urged the establishment, by Government, of Preparatory Medical Schools, which he thought might be readily accomplished; and in proof of which, and of the general excellence of such system, instanced the University of Michigan, which is of this character, and has carried out many suggestions of this Association. The report was received with applause, and the resolutions accompanying it were adopted.

Dr. Smith, of New York, Chairman of the Committee on Voluntary Essays, reported fifteen received, and premiums were awarded to the authors of the two following:

"On the Cell, its Physiology, Pathology," &c., by Dr. Waldo G. Burnett, of Boston.

"Fibrous Diseases of the Uterus, hitherto considered incurable," by Washington L. Atlee, of Philadelphia.

Dr. March, of Albany, then presented a paper on "Morbus Coxarius," or Hip Joint Disease.

On motion of Dr. Smith, Dr. March was requested to read his paper in the Crosby-st School, and to illustrate it by his specimens to-day at noon, on the adjournment of the meeting.

The following resolution was proposed by Professor Palmer, of Chicago:

*Resolved*, That this Association earnestly recommends to the local societies in different portions of our country, to appoint committees whose duties it shall be to record the prevalence of epidemic or other diseases, and the general state of health in their respective localities and transmit the said reports to the Committee of this body on Epidemics through the State Societies, where they exist.

Which resolution, together with another instructing the Secretary to circulate the above resolution through every State in the Union, was adopted.

On motion, the Association took a recess till 1½ o'clock.

AFTERNOON SESSION.

The Association was called to order by the President at 1½ o'clock.

The Secretary read an invitation to the members of this Association from the authorities of the New York Hospital to visit that institution.

The Secretary then read the names of a number of members who had just presented their credentials.

Dr. Charles A. Lee then offered the following resolutions:

*Resolved*, That it be adopted as the sense of this convention, that those colleges which give two courses of lectures annually, thereby making two colleges out of one, are unworthy of the commendation of this convention.

*Resolved*, That no person shall be received by this association, from any medical college which gives two courses of lectures annually, each counting towards a degree.

Dr. Morgan, from Washington, moved that the above resolutions be referred to the Committee on Education, of next year.

Moved by Dr. Atlee, of Philadelphia, that the above resolutions be laid upon the table. Carried.

A resolution was then carried, tendering the thanks of this convention to Doctor Winslow, of Boston, for the distinguished services he has rendered to the medical profession in the Legislature of Massachusetts.

Dr. Buck, of New York, then read a paper on morbid growths in the larynx. The natural specimen was exhibited, and also a wax model of the same. This paper was listened to with great interest by the Association. Dr. Buck detailed the various operations that he had performed on the patient, a widow lady of middle age, which were, unfortunately, unsuccessful.

Dr. Thomas G. Symons, Chairman of the Committee to memorialize Congress, to have the Medical Statistics of the United States printed separately for the use of the Medical Profession, reported.

The same gentleman presented the following report:

The undersigned, Chairman of a Committee appointed by the American Medical Association to memorialize Congress upon the importance of enacting, as an act of duty and humanity, a law making it obligatory upon all vessels conveying emigrants to have a regular surgeon on board, respectfully report:

That a memorial, signed by all the members of the Committee, the President and Secretaries of the Association, was, through the medium of Hon. A. P. Butler, Senator from South Carolina, presented in May, 1852, to the Senate of the United States, and was referred to the Committee on Commerce.

It is with regret that the undersigned has to report, that the Committee of Commerce, having, it is to be presumed, other subjects of much greater import in a pecuniary point of view, but assuredly not in a humane and philanthropic one, made no report.

It was not contemplated that any action would have been taken on the subject, at the late period of the session of 1852, but sanguine hopes were entertained that some report would have been made in the session of 1853. The undersigned, in common with every good citizen, has a respectful consideration for the legislature and public functionaries, but must honestly confess that the legislation growing out of untoward pressure for ulterior purposes of individual or political agrandizement, renders it difficult to have such prompt and efficient action as should be reasonably expected upon questions involving scientific advancement, or objects of Hygienic improvement. The undersigned, however, is fully impressed that no intentional disrespect was meant by the Committee on Commerce, in allowing the memorial to remain in their archives unnoticed, and now that the Presidential election and collateral subjects incidental, or having a bearing thereon are settled, it is but reasonable to suppose the Senate and House of Representatives would consider any measure as regards science, or medicine, or Hygiene, recommended by the American Medical Association, and likewise, if possible, in legislation, adopt the same. There has been every assurance given to the undersigned that the measure will have a proper consideration at the next Session of Congress, therefore he asks that the Committee be continued, and considered as reporting progress. All of which is respectfully submitted.

THO. G. SYMONS, M. D. Chairman.

Both of the above reports were accepted, and the Committee continued.

Dr. Peaslee, of New Hampshire, offered the following resolution, which he accompanied with a few brief and appropriate remarks:

*Resolved*, That it is the duty of the faculties to refuse to admit to examination, for the degree of Doctor, all persons who intend to engage in any other than the regular practice, and to give notice of this in their annual course of lectures.

Dr. Sayre said he thought the resolution was not calculated to effect the object it had in view. The best way, in his opinion, was to withdraw the diploma after it had been given, and in the event of its being used for the advancement of quackery. In conclusion, he moved the following resolution as an amendment:

*Resolved*, That a committee be appointed to memorialize the several State governments, in reference to the subject of diplomas to medical men, and to petition them, in the name of the Association for the passage of a law granting to chartered medical colleges the privilege of retracting publicly the diplomas of any of their graduates, when, in the judgment of the medical faculty of the college or school granting such diplomas or certificates, they may have forfeited a right to the same.

Dr. Gooch moved that the whole subject be laid upon the table to come up again before the meeting to-morrow. The motion was adopted, and the meeting soon after adjourned till 9 o'clock Thursday morning.

THIRD DAY.

The meeting of this body was re-opened pursuant to adjournment. Two hundred and seventy-six delegates were present, Dr. Knight, president, took the chair, and Dr. Beadle, Secretary, read the minutes of the proceedings on the previous day. Business was then resumed.

On motion of Dr. W. Hooker, a committee of five was appointed to take action in regard to the further organization of state and county medical societies.

Dr. Zeigler, of Pennsylvania, offered the following preamble and resolutions, which were referred to the committee on organizing state and county societies.

Inasmuch as the universal aggregation and organization of the members of the medical profession in the United States has long been a desideratum, but partially attainable until the adoption of the present mode of organziing local and general societies in imitation of, and in conformity with the confederacy of this country, and whereas, notwithstanding this consolidation has thus been more completely and perfectly effected, a very large portion of the profession still remain isolated and unassociated, thus retarding and preventing the more rapid attainment of those exalted objects involved in this universal professional union in one great brotherhood; and inasmuch as the present general mode of perfecting further and final professional aggregation and consolidation is comparatively imperfect, inefficient and protracting, and hence will require an almost indefinite period for its full accomplishment and final consummation and completion; and inasmuch as some more general and systematic effort for the speedy and positive realization of this highly important object is yet greatly desirable; therefore

*Resolved,* That the American Medical Association hereby reiterate the repeatedly previously expressed desire for the immediate formation and organization of county and state medical societies in every part of the country in which they have not yet been established.

*Resolved,* That every county medical society be and is hereby recommended to dispense with the present system of acquiring members by the previous pro formal manifestation of personal desire on the part of applicants for such association, and to substitute therefor that of the immediate and voluntary election to membership therein of every unassociated eligible physician.

*Resolved,* That all physicians thus voluntarily elected, and subsequently neglecting or declining to respond to and unite themselves with the general profession, shall be considered as estimating their own personal views, and private relations and interests, above and in opposition to those of the profession generally, and, as thus antagonistic to its exalted objects cannot therefore consistently expect the continued enjoyment of the usual rights and privileges of professional intercourse and fellowship.

Dr. N. S. Davis, of Chicago, read the report of the committee on medical literature, which was referred to the committee on publication. The document showed that there were twenty-eight periodicals of medical instruction and literature published in the Uuited States, which were issued at quarterly, monthly, semi-monthly, and weekly intervals. The committee had carefully examined their general contents and arrange ment previous to the years 1852, and from the April of that year to the month of March, 1853, and found that a considerable improvement had taken place. They enjoyed an aggregate circulation of about sixteen thousand, and contained a large amount of information in the shape of notices of lectures, communicated medical and surgical cases, obstetrics general hygiene, editorial matter, and reviews.

Dr. Yandell, of Kentucky, offered the the following resolutions which were unanimously adopted by a rising vote :

*Whereas*, By the dispensation of an inscrutable Providence, Dr. Daniel Drake has been removed since the last meeting of this Association from the scene of his earthly labors—

*Resolved*, That in the death of Dr. Drake the American Medical Association has lost one of its most valued members, and the American Medical profession one of its brightest ornaments.

*Resolved*, That his steady devotion to his profession through a long life, his zeal, activity, and unceasing efforts to advance its interests, afford an example worthy of the imitation of every young physician.

*Resolved*, That this Association will cherish the memory of Dr. Drake for his many virtues, and for his labors which have adorned and elevated our profession.

On motion of Dr. Condie, of Pennsylvania, it was

*Resolved*, That we have heard with sincere regret of the death of our late fellow member, Dr. Isaac Parrish, of Philadelphia, who was distinguished by his early and earnest advocacy of the establishment of this association, by his ardent interests in its proceedings, and by his valuable contributions to its publications.

On motion of Dr. Cox, of Maryland, it was also

*Resolved*, That in the demise of Dr. William E. Horner, which has occurred since the last annual session of this body, the American Medical Association has lost one of its illustrious and useful members; and the science of medicine an indefatigable student and most distin guished teacher.

*Resolved*, That his memory, dear as it must ever be to the lovers of medical science universally, will be especially cherished by this Association, to whose great objects and aims, his last efforts were during life, promptly and liberally bestowed.

On motion of Dr. Palmer, of Chicago the resolution offered yesterday from the Virginia Medical Society, recommending the appointment of a Chemist, and laid on the table, was taken up for consideration.

Dr. Parker, of Virginia, offered the following as an amendment:

*Resolved*, That this Association recommend Congress to consider the propriety of passing a law compelling all importers of nostrums to state upon all compounds thus imported their true constituents, and in English.

*Resolved*, That the Secretary be instructed to forward a copy of these resolutions to the Executive of the general Government.

An amendment to strike out all after the word "English," was accepted.

The first resolution was read by the Secretary.

Dr. Bond, of Baltimore, objected to the resolution, because it could do no good, and was likely to do a great deal of injury. He moved to lay on the table, but withdrew it to allow discussion.

Dr. Hooker, of Connecticut, said the facts from the State of Maine would answer the objections of Dr. Bond. An act had been passed there which was found to be a death-blow to this system of quackery. But on petition of the people and clergy, who were in favor of encouraging it, the law was repealed. He wished parties to know what they swallowed.

Dr. Sayre, of New York, said that every notice that was taken of any such quackeries only tended to advertise them, and could accomplish no useful purpose. He proposed to lay on the table. Lost.

Dr. Cox, of Maryland, never knew any good to follow prohibitory measures against quackery. In the State of Maryland a set of quacks known as Thompsonians, flourished under prohibitory legislature, and died away when given every freedom and latitude. He believed the surest death blow to quackery was to treat it with contempt and silence. It was unworthy of any grave notice from this Association. He believed the true remedy against quackery was to be found in attention to their Association.

Dr. Bolton, of Virginia, drew the attention of the Association to a resolution passed yesterday, thanking Dr. Winslow Lewis for his efforts in supporting a bill before the Massachusetts Legislature. There was a mistake in thinking that the resolution proposed to prohibit the sale of these quack medicines; it only proposed that the ingredients of which they were composed should be set forth on the label. This would divest the medicine of the great charm of mystery which gave them such importance in the minds of the public. They would see that for a few pence they could procure some simple medicine, which was the chief ingredient in the expensive bottle.

Dr. Richards, of Ohio, and Dr. Jackson, of Massachusetts, followed in opposition to the resolution. The analyzing of these nostrums, by order of the State, would be giving a sanction to their sale.

The reading of the resolution was called for, and a vote taken.

President.—The Nays are the loudest; whether they are the more numerous, I cannot say.

The House was counted, and the resolution laid on the table.

Dr. Yandell presented a report received from Dr. S. D. Gross, on the results of Surgical operations for the relief of malignant diseases, which was referred to a Committee on Publication. Dr Y. read a brief abstract, as follows:

From the facts and statements which have now been presented, embracing the opinions of many of the most intelligent, experienced and distinguished practitioners in different ages, and in different parts of the world, the following conclusions may be legitimately deduced:

*First.*—That cancerous affections, particularly those of the mammary gland, have always, with a few rare exceptions, been regarded by practitioners as incurable by the knife and escharotics. This opinion, commencing with Hippocrates, the father of medicine, has prevailed from the earliest records of the profession, to the present moment. Nature never cures a disease of the kind; nor can this be effected by any medicine, or internal remedies, known to the profession.

*Secondly.*—That excision, however early and thoroughly executed is nearly always, in genuine cancer, followed by relapse, at a period varying from a few weeks to several months, from the time of the operation.

*Thirdly.*—That nearly all practitioners, from the time of Hippocrates to the present day, have been, and are still averse to any operation for the removal of cancerous tumors, after the establishment of ulceracion, rapid growth, firm adhesion, organic change in the skin, lymphatic invasion, the cancerous dyscrasy, or serious constitutional derangements; on the ground that, if had recourse to, under these circumstances, the malady almost inevitably recurs in a very short time, and frequently destroys the patient more rapidly than when it is permitted to pursue its own course.

*Fourthly.*—That in all cases of *acute* car[illegible]oma, or, in other words, in all cases of this disease, attended with very rapid development and great bulk of the tumor, extirpation is improper and unjustifiable, inasmuch as it will only tend to expedite the fatal result, which, under such circumstances, always takes place in a very short time.

*Fifthly.*—That all operations performed for the removal of encephaloid cancer and its different varieties, are more certainly followed by rapid relapse than operations performed upon schirrus or hard cancer.

*Sixthly.*—That in nearly all the operations for cancerous diseases, hitherto reported, the history has been imperfectly presented, being deficient in the details which are necessary to a complete and thorough understanding of the subject in each case. This remark is particularly true in reference to the diagnosis of the malady, the minute examination of the morbid structure, and the history of the case after the operation, as to the period of relapse, the time and nature of the patient's death, and the result of the post mortem examination.

*Seventhly.*—That cancerous affections of the lip and skin, now usually described under the name of cancroid diseases, are less liable to relapse after exterpation than genuine cancerous maladies, or those which are characterized by the existence of the true cancer cell and cancer-juice.

*Eighthly.*—That, although practitioners have always been aware, from the earliest professional records, of the great liability of cancer to relapse after extirpation, a great majority of them have always been, and still are, in favor of operation in the early stage of the disease, especially in schirrus, before the tumor has made much progress, or before there is any disease of the lymphatic ganglions, or evidence of the cancerous cachexy.

*Ninthly.*—That many cases of tumors, especially tumors of the breast and testicles, supposed to be cancerous, are in reality not cancerous, but of a benign character, and consequently, readily curable by ablation, whether effected by the knife or by escharotics. It is to this circumstance that we must ascribe the astonishing success which is said to have attended the practice of Hill, of Scotland, Nooth, of England, and Flajani, of Italy.

*Tenthly.*—That all operators insist upon the most thorough excision possible; removing not merely the diseased mass, but also a portion of the surrounding and apparently healthy tissues, as well as all enlarged and indurated ganglions.

*Eleventhly.*—That the practice has always prevailed, and still obtains, to save, if possible, a sufficient amount of healthy integument to cover the wound, and to unite, if possible, the wound by the first intention; on the ground that these precautions will tend much to retard, if not to prevent a recurrence of the disease.

*Twelfthly.*—That much stress is laid by writers upon a properly regulated diet, aud attention to the bowels and secretions after operation, as means of retarding and preventing relapse.

*Thirteenthly.*—That there is no remedy, medicine or method of treatment which has the power, so far as we are enabled to judge of its virtues, of preventing the reproduction of the morbid action after operation, no matter how early or how thoroughly it may be performed.

*Fourteenthly.*—That life has occasionally been prolonged and even saved by operation after relapse, as in some of the remarkable cases mentioned in a previous part of this report; but that, as a general rule, such a procedure is as incompetent to effect a permanent cure as a first extirpation.

Dr. Bryan, of Pennsylvania, rose to correct a statement made yesterday, by Prof. Jackson, that a young gentleman had been allowed to graduate in Philadelphia Medical College, after two weeks study.

Prof. Jackson, stated that he had received the information from a gentleman, whom he named, and had used it as an illustration of his argument.

Dr. Gooch, called up the subject of the graduating pledge, proposed by Dr. Peaslee, of New Hampshire, last evening, and laid over, and proposed the following resolutions:

*Resolved*, That this Association earnestly recommends to all the respectable Medical Colleges of the United States to administer to their graduates, previous to receiving the diploma, some pledge that they will maintain, to the best of their abilities, the honor and dignity of the profession; and that they will forfeit their degrees, whenever they desert the orthodox system of medicine.

*Resolved*, That the schools be urged not to graduate any man without requiring him to read the National Code of Ethics and publicly give his consent to abide by it, and that they will reserve to themselves the right to withdraw the diploma, publicly, whenever *the graduating* pledge has been violated.

Dr. John H. Phillips, of New Jersey, offered the following amendment:

*Resolved*, That it is the duty of all Boards of Examiners, to whom candidates may apply for examination or approval, to admit none but those who give satisfactory evidence of a good preliminary education, and that a regular Course of Medical Practice will afterwards be pursued and who shall subscribe to the Code of Ethics adopted by this Association.

Dr. Cox, of Maryland, thought the resolution contemplated an extraordinary act of legislation, and there would be great difficulty in applying the principle. The power of revoking a diploma, once given under the legal sanction of a charter, was a dangerous one to be intrusted to any set of men,

Dr. Atkinson, of Virginia, thought, that until the millennium, quackery would exist in the profession to some extent, and it was vain to legislate against it.

On motion of Dr. Sayre, of New York, the Association took a recess for one hour, without disposing of the resolution.

AFTERNOON SESSION.

The Association was called to order at 1½ P. M.

The consideration of Dr. Gooch's resolution was resumed.

Dr. Stillé, of Pennsylvania, offered the following:

*Resolved*, That in order to preserve the purity and honor of the medical profession, and to place around young practitioners an additional safe-guard against temptations to do wrong, as well as to draw a more distinct line between true and false physicians—it is hereby recommended to the several Medical Colleges, and such other Boards as are by law authorized to examine candidates for admission into the medical profession, to require from every graduate or licentiate, his signature to the Code of Ethics of this Association, as well as to furnish him with a copy of the Code, and it is also recommended that the formal administration of a pledge, faithfully to observe and keep the same, form part of the public ceremonies of Medical Commencement.

After some discussion the whole subject was referred to a Committee of three, to report as soon as possible.

Dr. Blatchford, of New York, moved to have a resolution offered yesterday, on the licensing power, with amendments by Dr. Garnett, taken up for consideration,

Dr. Hooker, of Connecticut, spoke to the subject of the resolutions. He would expose individual cases, without referring to the Colleges or individuals, by name. The object of such an Association as this, was not to be personal, but to reform abuses. Dr. H. cited several cases, for which he stated he had the best authority, in which young men had been allowed to graduate after terms of study under one year. Four of these cases, were from schools very prominent in the community. If such instances as he had stated had come without inquiry, was it not probable that if a searching inquiry were instituted numerous such abuses would be discovered. The cause of this was in a great degree owing to the competition between schools. He wished that the subject should be brought before a Committee; and he thought the remedy proposed in Dr. Garnett's amendment would prove efficacious.

Dr. Johnson, of St, Louis, was happy the subject had come before this Association. He was sorry the gentleman had not given the instances he referred to; for he was confident that the Association was composed of gentlemen who would have an influence in preventing such abuses. In Missouri, they had memorialized the Legislature, and found that no hope for any measure to protect the Medical Faculty could be expected from the Legislature. This Association would in itself have a much greater influence, by denying those schools which acted in the manner described, a place on this floor. The moral power of this Association, if exercised, would have the effect of keeping members of the profession from such practices.

Dr. Atlee, of Pennsylvania, rose to prevent a discussion on the subject, which could lead to no practical good for the present. The evil was a very great one, and he hoped the whole subject would be referred to a committee to make their report at the next meeting.

The subject was referred to the following Committee: Drs. Samuel Jackson, T. Blatchford, Johnson, of Missouri, and Peaslee of New Hampshire.

On motion of Dr. Atlee, the subject of proposed amendments to the Constitution was taken up, and the original articles, with proposed amendments read by the Secretary.

Dr. Stevens, of New York, moved an indefinite postponement of the whole subject of amendments to the Constitution.

Dr. Atkinson, of Virginia, moved to have the amendment proposing to admit four delegates from the United States Army and United States Navy, excepted from motion to postpone.

Dr. Coolidge, of United States Army, hoped the Association would decide whether delegates from the Army and Navy were entitled to a seat on the same footing as other delegates. If the Association did not do so, he thought the Chief of the Army and Navy Medical Bureau would not be inclined to nominate delegates from that department in future.

Dr. Bolton, of Virginia, read some resolutions intrusted to him by the Medical Society of Virginia, on the subject of amending the Constitution.

Dr. Stewart said the only alterations proposed were, in effect, to have only one delegate from each college, and one from each hospital, instead of two. This was that they might obtain, as nearly as posrible a uniform representation of ten per cent., from all branches of the profession. Also the change respecting the Army and Navy delegates.

The question on indefinite postponement, excepting the clause altering the Constitution in relation to delegates from the Army and Navy was put and carried, and the following adopted:

*Resolved*, That the second clause of Article 2 of the Constitution be so amended as to admit the American Medical Society in Paris to representation in this body upon the same terms as this Medcial bodies in the country.

Dr. Alfred Stille, Chairman of the Committee to whom was referred sundry memorials touching the course to be pursued by Medical Colleges and other Boards in the examination of candidates and the granting of Diplomas reported, submitting the following resolutions for adoption :

*Resolved*, That in order to preserve the purity and honor of the medical profession, and to place around young practitioners additional safe-guards against temptations to do wrong, as well as to draw a more distinct line of separation between true and false physicians, it be and is hereby recommended, that every graduate in Medicine be required to subscribe a pledge to submit to the revocation of his diploma upon conviction of having knowingly violated the Code of Ethics of this Aisociation. It is also recommended to the several Medical Colleges and such other Boards as are by law authorized to examine candidates for admission into the Medical Profession, to require from every graduate or licentiate his signature to the Code of Ethics of this Association, and to furnish him with a copy of the same. It is further recommended that the formal administration of a pledge faithfully to observe and keep the said Code form part of the public exercises of Medical Commencements.

The following form of Promise was among the documents referred to the Committee on Pledge:

I, A. B., of ———, in the State of ———, do hereby promise, on the honor of a gentleman, that I will conform strictly to the Code of Ethics of this my Alma Mater in all things pertaining to the practice of my profession; and when I shall fail to do so, I hereby grant to the Faculty of said School full power and authority to withdraw said Diploma, and all the rights and privileges which it is intended to confer.

Dr. Palmer, of Ills., and other delegates opposed that part of the report proposing to clothe colleges with the power of revoking diplomas for a breach of the "Code of Ethics."

Several motions and countermotions were made. The Chairman decided on the right of the Committee to withdraw the objectionable resolution, when the second and third recommendations of the report were adopted.

Dr. Sayre, of New-York, moved that the resolution be taken up, and passed as the sense of the meeting. It was taken up, and referred to Committee.

Dr. Palmer moved the following, which was adopted:

*Resolved*, That the Standing Committee, of which Dr. Bolton is Chairman, be instructed to inquire into all cases of death that may be reported as occurring from the use of anæsthetic agents during the present year in the United States, and report to the next meeting of the Association.

Dr. J. M. Smith, of New York, read the following

REPORT OF COMMITTEE ON NOMINATIONS.

The Committee on Nominations, in fulfilling the duty of their appointment, propose to continue most of the Special Committees appointed by the Association, in May, 1851, and May, 1852, and to appoint several new Special Committees They, therefore, submit the following list of Chairmen of Special Committees, with the subjects to them committed:

1, Dr. D. F. Condie, o Philadelphia, Penn., "On the Causes of Tubercular Disease."

2. Dr. James Jones, o New Orleans, La., "On the Mutual Relations of Yellow and Bilious Remittent Fever."

3. Dr. R. S. Holmes, of St. Louis, Mo., "On Epidemic Erysipelas."
4. Dr. Geo. B. Wood, of Philadelphia, Penn., "On Diseases of Parasitic Origin."
5. Dr. R. D. Arnold, of Savannah, Ga., "On the Physiological Peculiarities and Diseases of Negroes."
6. Dr. James R. Wood, of New York, "On Statistics of the operation for the removal of Stone in the Bladder."
7. Dr. F. Peyre Porcher, of Charleston, S. C.—"On Toxicological and Medicinal Properties of our Cryptogamic Plants."
8. Dr. Goodrich A. Wilson, of Virginia—"On Cholera and its Relation to Congestive Fever—their Analogy or Identity."
9. Dr. Worthington Hooker, of Connecticut—"On Epidemics of New England and New-York."
10. Dr. John L. Attee, of Lancaster, Penn.—"On Epidemics of New-Jersey, Pennsylvania, Delaware and Maryland."
11. Dr. D. G. Cain, of Charleston, S. C.—"On Epidemics of Mississippi, Louisiana, Texas and Arkansas."
12. Dr. W. L. Sutton, of Georgetown, Ky.—"On Epidemics of Tennessee and Kentucky."
13. Dr. Thomas Keyburn, of St. Louis Mo.—"On Epidemics of Missouri, Illinois, Iowa and Wisconsin."
14. Dr. George Mendenhall, of Cincinnati, Ohio—"On Epidemics of Ohio, Indiana and Michigan."
15. Dr. E. D. Fenner, of New Orleans, La.—"On Epidemics of Mississippi, Louisiana, Texas and Arkansas."
16. Dr. Chas. A. Lee, of New York—"On Domestic Hygiene."
17. Dr. Daniel Brainard, of Chicago, Ill.—"On the Constitutional and Local Trearment of Carcinoma."
18. Dr. N. S. Davis, of Chicago, Ill.—"On the Influence of Local Circumstances on the Origin and Prevalence of Typhoid Fever."
19. Dr. Geo. Engelman, of St. Louis, Mo.—"On the Influence of Geological Formation on the Character of Disease."
20. Dr Henry M. Bullitt, of Louisvslle, Ky.—"On the Use and Effect of Applications of Nitrate of Silver to the Throat, either in Local or General Disease."
21. Dr. Robert Campbell, of Augusta, Ga.—"On the Pathogenic Influence of Feather Beds."
22. Dr. James Bolton, of Richmond, Va.—"On the Adminisiration of Anæsthetic Agents during Parturition."
23. Dr. Henry Taylor, of Mount Clemens, Mich.—"On Dysentery."
24. Dr. F. Donaldson, of Baltimore, Md—"On the Present and Prospective value of the Microscope in Disease."
25. Dr. R. L. Howard, of Columbus, Ohio—"On the Pathology and Treatment of Scrofula."

*Committee on Plans of Organization for State and Cvunty Societies.*—Isaac Hays, M D., of Pennsylvania, Chairman; Worthington Hooker, M. D, of Connecticut; Josiah Andrews, M. D., of Michigan;

B. R. Wellford, M. D., of Virginia; A. L. Pierson, M. D., of Massachusetts.

*Committee on Medical Literature.*—T. S. Bell, M. D., of Kentucky, Chairman; Samuel H Pennington, M. D., of New-Jersey; Ed. H. Parker, M. D., of New Hampshire; William K. Bowling, M. D., of Tennessee; Zina Pitcher, M. D, of Michigan.

*Committee on Medical Education.*—B. R. Wellford, M. D., of Virginia, Chairman; Resign Lowe, M. D., of Iowa; Lynden A. Smith, M. D., of New Jersey; Jacob Bigelow, M. D., of Massachusetts; L. A. Dugas, M. D., of Georgia.

*Committee on Volunteer Communications.*—Drs. C. A. Pope, Thos. Reyburn, John S. Moore, J. B. Johnson and A. Litton, of St. Louis, Mo.

*Committee of Arrangements.*—Drs. J. R. Washington, J. S. Moore, S. Pollok, Thos. Reyburn, J. O'Farrar, W. M. McPheeters, C. W. Hempstead and E. S. Lemoine, of St. Louis Mo.

*Committee on Publications.*—Dr. D. F. Condie, Pennsylvania, Chairman; Dr. E. L. Beadle, of New-York; Dr. A. Stille, Pennsylvania; Dr. I. Hays, Pennsylvania; Dr. E. S. Lemoine, of Missouri; Dr. G. Emmerson, Pennsylvania; Dr. G. W. Norris, Pennsylvania.

Dr. Atlee, of Pennsylvania, moved that when the meeting adjourn it adjourn to meet at 9 o'clock on Saturday morning.

The motion was withdrawn.

Dr. Roddey, of Virginia, moved to have the report and resolutions offered by Dr. Zeigler, and laid on the table, taken up.

The report and resolutions were referred to a committee consisting of Drs. Zeigler, Roddey and Jackson.

Drs. Atlee and Millenberger offered the following, which was adopted

*Resolved,* That the cordial thanks of the American Medical Association be, and they are hereby tendered to the Committee of Arrangements, the Trustees of the Church in which they have held their meetings, the profession and the citizens generally of New-York, for the generous and elegant hospitality extended towards its members during its present session.

Dr. Bolton proposed a vote of thanks to the President of the Institutions which had been thrown open for members of the Association during their stay.

Dr. Bolton, of Virginia, moved the thanks of the Association to the Press of the City, for its accurate reports of their proceedings.

On motion, it was resolved to meet on board the steamboat, at foot of Pier No. 3, Friday morning, at 9 o'clock, and proceed to visit the public institutions belonging to the City of New-York.

The President congratulated members on the close of their deliberations, and expressed his wish that they should all meet at St. Louis next year.

The Association then adjourned *sine die.*

In the evening after the adjournment of the Association the members were invited to a sumptuous dinner provided by the Physicians of New York. The entertainment was superb; it was intended to be the most magnificient ever prepared in our country, and we presume the effort was successful. The room, the dinner itself, the galleries, filled with the beauty and fashion of New York, presented a spectacle which those who beheld it will never forget.

We had hoped to conclude our account of this interesting meeting in this number but find we have not space—it will be completed in our next.

